# Liquid crystal phases of two-dimensional dipolar gases and Berezinskii-Kosterlitz-Thouless melting

**DOI:** 10.1038/srep19038

**Published:** 2016-01-11

**Authors:** Zhigang Wu, Jens K. Block, Georg M. Bruun

**Affiliations:** 1 Aarhus University, Department of Physics and Astronomy, Aarhus C, DK-8000, Denmark

## Abstract

Liquid crystals are phases of matter intermediate between crystals and liquids. Whereas classical liquid crystals have been known for a long time and are used in electro-optical displays, much less is known about their quantum counterparts. There is growing evidence that quantum liquid crystals play a central role in many electron systems including high temperature superconductors, but a quantitative understanding is lacking due to disorder and other complications. Here, we analyse the quantum phase diagram of a two-dimensional dipolar gas, which exhibits stripe, nematic and supersolid phases. We calculate the stiffness constants determining the stability of the nematic and stripe phases, and the melting of the stripes set by the proliferation of topological defects is analysed microscopically. Our results for the critical temperatures of these phases demonstrate that a controlled study of the interplay between quantum liquid and superfluid phases is within experimental reach for the first time, using dipolar gases.

With the impressive experimental progress in trapping and cooling gases consisting of dipolar atoms and molecules, a new and very promising research field is emerging. Since the early experiments trapping bosonic Cr atoms with a large magnetic dipole moment^1^, fermionic Dy and Er atoms^2^,^3^ are now being trapped and cooled to degeneracy, and one has already observed Fermi surface deformation due to the dipolar interaction^4^. Following the ground breaking experiments trapping KRb molecules with an electric dipole moment^5^,^6^,^7^, fermionic LiCs^8^, NaLi^9^, and NaK^10^ molecules with a large electric dipole moment are now being trapped. The reason for this intense experimental activity is that the anisotropic and long range nature of the dipole-dipole interaction is predicted to give rise to several exotic forms of matter, many of which have never been realised before in nature^11^,^12^,^13^.

A system of particular focus in this research field is a two-dimensional (2D) dipolar Fermi gas. This system is predicted to exhibit a range of intriguing phases at zero temperature 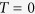, including striped (smectic)^14^,^15^,^16^,^17^,^18^,^19^, *p*-wave superfluid^20^, supersolid^21^, hexatic^22^,^23^, and Wigner crystal phases^24^,^25^. Here, we analyse the properties of this system for 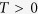. This includes the melting of the striped phase, whose low energy degrees of freedom are described by an anisotropic XY model. We determine the stiffness constants of this effective model microscopically. The melting is driven by the proliferation of topological defects called dislocations, and the corresponding Berezinskii-Kosterlitz-Thouless (BKT) critical temperature is determined by the well-known renormalisation group equations. For large tilting angles of the dipoles, the system can have additional superfluid pairing which coexists with the stripe order, and we calculate the critical temperature of the superfluid transition. When the dipoles are perpendicular to the 2D plane, the critical temperature of the stripe phase is shown to vanish, and the system exhibits a nematic phase characterised by long range orientational order but no translational order.

Our results demonstrate that it is within experimental reach to realise quantum liquid crystal and superfluid phases with dipolar gases. This makes it possible to systematically investigate the interplay between spontaneously broken translational, rotational, and gauge symmetries, which is believed to play an important role in many of the most interesting electronic materials discovered in recent decades^26^,^27^,^28^,^29^,^30^. Moreover, our results show that one can confirm the microscopic mechanism behind the BKT transition, namely the proliferation of topological defects, simply by observing the proliferation of dislocation defects in the stripe pattern. Whereas early experiments reported only indirect evidence of BKT physics in the bulk properties^31^,^32^,^33^,^34^,^35^,^36^,^37^,^38^, tremendous progress has recently been achieved in probing the microscopic aspects of the BKT transition using cold atoms^39^,^40^,^41^.

## Results

We consider fermionic dipoles of mass *m* and average areal density 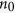, which are restricted to move in the 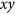 plane by a tight harmonic trapping potential 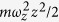 along the *z*-direction. In the limit 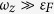, where 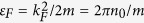 is the Fermi energy of a 2D non-interacting gas with areal density 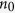, the system is effectively 2D with the dipoles frozen in the harmonic oscillator ground state in the *z* direction. An external field aligns the dipoles so that their dipole moment d is perpendicular to the *y*-axis and forms an angle 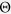 with the *z*-axis. The dipole-dipole interaction is 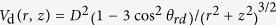, where 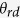 is the angle between the relative displacement vector of the two dipoles 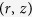 with 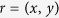 and the dipole moment d, and 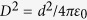 for electric dipoles and 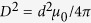 for magnetic ones.

The strength of the interaction is determined by the dimensionless parameter 
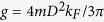
, and the degree of anisotropy is controlled by the tilting angle 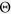. The system is rotationally symmetric for 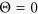 and becomes more anisotropic with increasing 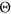. Above a critical interaction strength 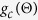, it is predicted to form density stripes at 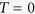, where the density exhibits periodic modulations of the form


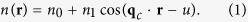


Here, 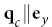 is the wave vector of the stripes, and 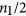 and *u* are, respectively, the amplitude and the phase of the complex stripe order parameter 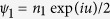. The density modulation is formed along the *y*-direction so as to minimise the interaction energy. The system thus exhibits liquid-like correlations along the *x*-direction and crystalline correlations along the *y*-direction. This phase has been predicted by Hartree-Fock theory^14^,^15^,^16^,^17^,^42^, density-functional theory^19^, and by a variant of the so-called STLS method^18^. Remarkably, Hartree-Fock and density-functional theory predict essentially the same critical coupling strength 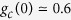 for stripe formation at 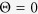, whereas the STLS method obtains a somewhat higher value. In general, since the formation of stripes occurs at relatively strong coupling it is difficult to make quantitatively accurate predictions, and one will probably have to wait for experiments to sort out the precise physics. For 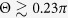, the system is predicted to become a *p*-wave superfluid^20^, which for strong enough coupling can coexist with the stripe order forming a supersolid^21^. Quantum Monte Carlo simulations also predict the dipoles to form a triangular Wigner crystal at very large values of 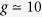 for 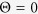
24,25 in analogy with the case of bosonic dipoles^43^,^44^,^45^. This very strong coupling regime is outside the scope of the present paper.

### Stripe phase at finite *T* and effective XY model

Since the stripe phase breaks translational invariance along the *y*-direction, it is a quantum analog of a classical smectic liquid crystal^46^,^47^. Indeed, the system has a manifold of equivalent ground states distinguished only by a constant factor *u*, which specifies the position of the stripes along the *y*-direction. Consequently, there are low energy collective excitations associated with a spatially dependent phase 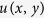. Moreover, since a change from *u* to 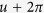 returns the system to the same ground state, it follows that the low energy degrees of freedom of the stripe phase are described by a 2D anisotropic XY model. Specifically, the simplest form of the elastic free energy congruent with the symmetry of the system is given by


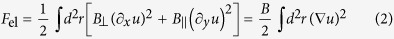


for 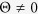. Here, 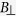 and 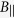 are the perpendicular and parallel elastic coefficients describing respectively the energy cost of small rotations and compressions/expansions the stripes. In the second equality, we have used the rescaling 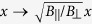 to obtain an isotropic XY model with the effective elastic constant 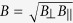.

### Berezinskii-Kosterlitz-Thouless melting

**Figure 1:**
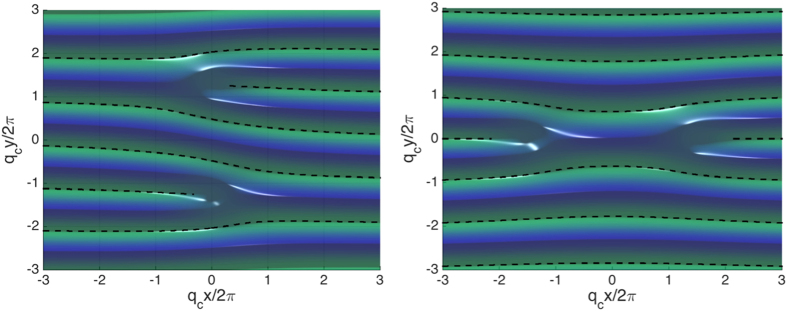
Dislocation pairs in the stripe phase. The dislocations are centered at 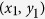 and 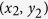 so that the phase field is 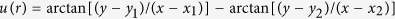. Left: A 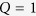 dislocation centered at 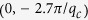 and a 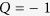 dislocation centered at 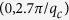. Right: A 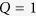 dislocation centered at 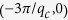 and a 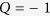 dislocation centered at 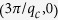. The dashed lines indicate the position of the density maxima.

As the stripe phase is described by the XY model, it exhibits algebraic long-range order at sufficiently low temperatures and it melts via the Berezinskii-Kosterlitz-Thouless mechanism due to the proliferation of topological defects^48^,^49^,^50^,^51^. In the case of the stripe phase, the topological defects are dislocations. The phase field for a single dislocation of charge 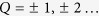 satisfies 
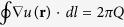
, where the path of the integration encloses the core of the dislocation. The presence of such a dislocation corresponds to inserting *Q* extra stripes to the left (right) of the dislocation for 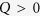 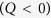. The energy of a single dislocation consists of a core part 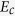, and a part that scales logarithmically with the size of the system. Pairs of bound dislocations with opposite charges 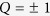 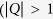 are energetically suppressed), however, have a finite energy even for an infinite system size and can be thermally excited in the stripe phase. This is due to the fact that the phase fields of the oppositely-charged dislocations cancel at large distances, which results in merely a local disturbance of the density. In Fig. 1, we illustrate dislocation pairs with opposite charges 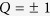 centered at 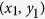 and 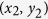 respectively. The stripe amplitude is suppressed in the core regions of the defects due to the large energy cost associated with 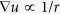, where *r* is the distance to the core. From the rescaling 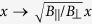 it follows that the energy of a vertically displaced dislocation pair distance *δ* apart is the same as that of a pair displaced horizontally by the distance 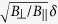. Since 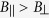 as we will demonstrate below, this shows that the dislocation pairs along the *x*-direction are more tightly bound than those along the *y*-direction.

The spontaneous thermal excitation of bound dislocation pairs decreases the elastic coefficients at a macroscopic scale. The softening of the effective stiffness constant *B* can be calculated from the well-known renormalisation group equations as described in the methods section. At a critical temperature 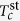, the renormalised elastic coefficient 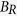 drops to zero by a sudden jump of magnitude 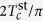. This disappearance of elastic rigidity signals the melting of the density stripes.

### Calculation of bare stiffness constants

**Figure 2:**
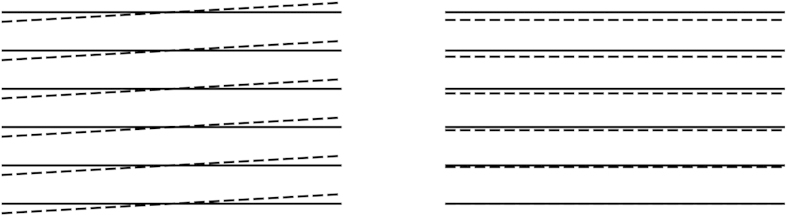
Elementary distortions of the stripes. Rotation (left) and compression (right) of the stripes away from their equilibrium positions indicated by the solid lines.

We now turn to a microscopic calculation of the “bare” stiffness constants 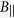 and 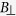 unrenormalised by dislocation pairs. The relevant thermodynamic quantity is the free energy of the system 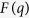, which depends on the stripe wave vector *q*. Any non-uniform phase fluctuation increases the free energy by an amount given by (2) for long wave lengths. To extract the elastic coefficients 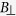 and 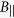, we consider two specific distortions: an infinitesimal rotation and an infinitesimal compression/expansion of the stripes away from the equilibrium configuration, as illustrated in Fig. 2.

These distortions are described by the phase field 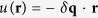, where 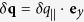 for the compression and 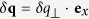 for the rotation. They are thus equivalent to a variation of the stripe vector 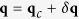. Inserting the phase fluctuations into Eq. (2), we obtain the increment of the free energy


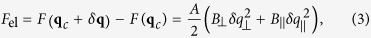


where *A* is the area of the system, and we have used the equilibrium condition 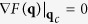. We thus find


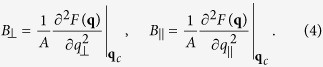


The interaction energy per particle due to stripe formation scales as 
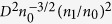
. Assuming that the interaction energy is dominant, we find that the elastic coefficient *B* scales as 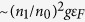 for a fixed 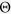. The magnitude of *B* can be further reduced by a geometrical factor depending on 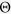, since the system becomes rotationally symmetric for 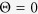, as we shall discuss below.

In order to microscopically calculate the bare stiffness constants, we employ Hartree-Fock mean-field theory for the free energy, writing 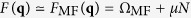, where 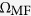 is the mean-field thermodynamic potential given by


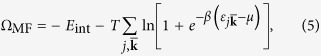


*μ* is the chemical potential and *N* is the total number of particles. The quasiparticle energies are 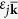, where 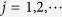 is the band index and 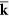 is restricted to the first Brillouin zone of the 1D periodic potential set up by the stripes. We subtract the interaction energy 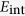 to avoid double counting. The details of this calculation are given in the methods section.

**Figure 3:**
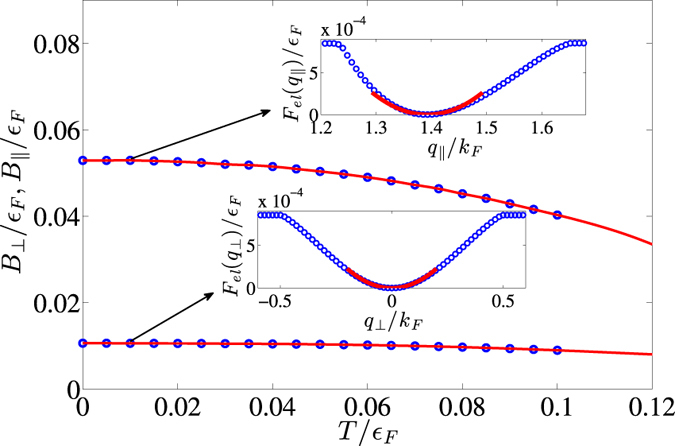
Bare stiffness constants as a function of temperature. Upper curve gives 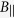 and lower curve 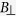 for 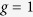 and 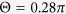. The blue circles in the insets are elastic free energy plotted as a function of *q* at 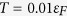, and the red solid curves are parabolic fits to several data points in the vicinity of 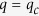.

In Fig. 3, we plot the bare elastic coefficients obtained from this approach as a function of temperature for 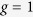 and 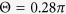, for which the system has a large stripe amplitude 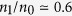 at low temperatures. In order to minimize finite size effects, we determine the elastic coefficients by fitting a parabolic curve to the free energy in the vicinity of 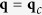 in accordance with (3), instead of performing a numerical differentiation following (4). This is illustrated in the insets of Fig. 3. This procedure allows us to obtain numerically accurate values for the elastic coefficients. From Fig. 3, we see that both elastic coefficients decrease with increased temperature. This is expected since thermal excitations of quasi-particles reduce the stripe amplitude and thus their rigidity. We also find that 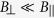, which suggests that compressing/expanding the stripes costs more energy than a rotation. This difference in magnitude becomes even more profound for small 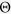 when 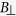 is strongly suppressed by the weak anisotropy of the system. Finally we note that for 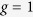 and 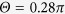, the system is in fact predicted to have additional superfluid pairing at 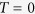
21. However, as demonstrated in ref. 21, the superfluid order has negligible effects on the stripe formation, and it can thus be safely neglected when analysing the elastic properties of the stripes.

**Figure 4:**
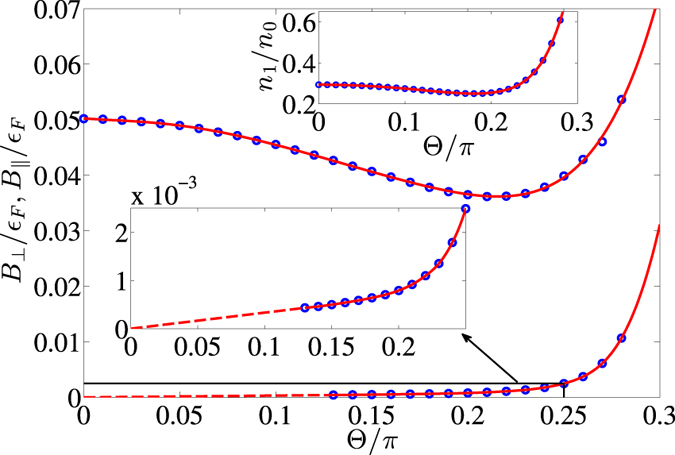
Bare stiffness constants as a function of tilting angle. Upper curve gives 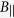 and lower curve 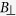 for 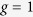 and 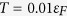. For 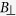, the red dashed line is an extrapolation for 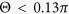, where the coefficient is too small to be accurately determined with our numerical method. The upper inset is a plot of the relative stripe amplitude as a function of 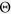. The lower inset is an expanded view of the 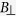 for small values of 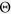.

In Fig. 4, we plot the bare elastic constants as a function of the tilting angle 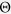 for 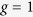 and 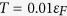. The elastic constant 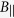 depends non-monotonically on 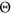, first decreasing and then increasing exhibiting a minimum at 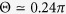. This is consistent with the mean-field phase diagram, which shows that the stripe formation is somewhat suppressed for intermediate values of 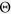
21,42. To illustrate this, we plot as an inset the stripe amplitude 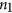 as a function of 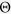; we see that it exhibits the same non-monotonic behaviour as 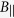. It is an intriguing question whether this non-monotonic behaviour is an artifact of the Hartree-Fock approximation or whether it is a real physical effect. In comparison to this behaviour, Fig. 4 shows that 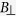 increases monotonically in 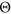. In particular, we have 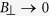 for 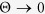 as shown in detail in the inset. This reflects that the system is rotationally symmetric for 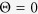 such that a rotation of the stripes costs no energy.

### Renormalised stiffness constants and stripe melting

**Figure 5:**
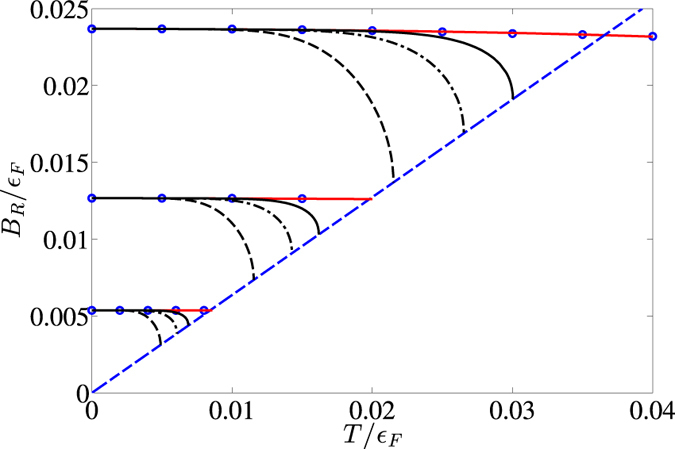
Renormalised stiffness constants as a function of temperature. The three groups of curves for 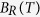 for 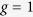 correspond to, in the order from bottom to up, 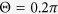, 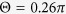 and 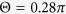 respectively. In each of the group, the four curves, in the order from bottom to up, correspond to 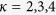 and 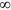 (mean-field result) respectively. The slope of the dashed diagonal line is 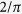.

The bare elastic constants obtained from the mean-field theory can now be used as initial values in the RG equations to determined the renormalised elastic constants. We also need the dislocation core energy, which must scale as 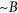. Therefore, we write 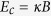, where 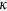 is a constant of order unity. In Fig. 5, we plot the renormalised elastic coefficient 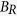 as a function of temperature, obtained by solving (16) with the initial mean-field values of 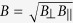 and 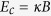 for various coupling strengths *g* and tilting angles 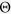. To examine the dependence on the core energy, we have chosen different values of 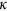. We see that the thermal excitation of dislocation pairs soften the elastic coefficients as expected. This softening is negligible for low *T* where the core energy prohibits the excitation of dislocations. The softening increases with decreasing core energy and increasing *T*. At the critical temperature 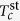 determined by the solution to 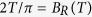, the elastic coefficient drops to zero discontinuously and the density stripes melt.

The temperature dependence of renormalised elastic coefficient 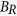 shown in Fig. 5 can be probed experimentally by a measurement of correlation function 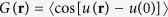 for the fluctuations in the stripe positions. This correlation function exhibits the characteristic scale invariant algebraic decay below the BKT transition temperature^46^


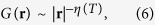


where 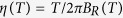. We have 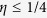, and it tends to the universal value of 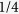 when 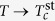.

**Figure 6:**
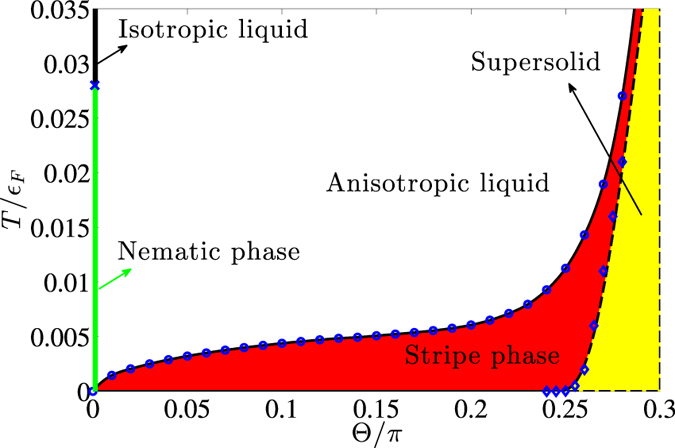
Phase diagram for *g* = 1. The system is in the stripe phase below the Kosterlitz-Thouless melting temperature which is calculated taking 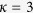 for the core energy. For 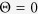, the striped phase melts at 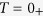 into a nematic phase with long range orientational order but no translational order. The nematic phase melts into an isotropic liquid at a temperature 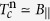 indicated by the blue cross. For 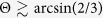 the system is in a supersolid phase at 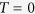 with both stripe and superfluid order. The transition temperatures of this phase calculated for various tilting angles are indicated by the diamonds. The dashed curve is a fit to the data by (8). The system collapses for 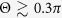.

The resulting melting temperature 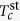 is plotted in Fig. 6 as a function of 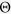 for 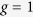 and 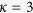. It increases rapidly with 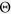, indicating that the degree of anisotropy of the system increases such that the stripes become more rigid. An extrapolation of our calculation for 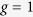 and 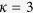 shows that 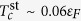 for 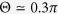. The critical temperature also increases with the coupling strength, scaling as 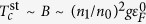. We note that in addition to the explicit linear dependence on *g*, the 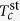 can further increase with the coupling strength through the dependence on 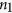. Our results show that in order to observe the stripe phase and the associated BKT physics with dipoles, it is preferable to choose a large tilting angle in addition to having a large dipole moment. However, the tilting angle cannot exceed 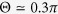 above which the system exhibits a density collapse for large coupling strengths^20^,^21^.

We note that the critical temperature increases with increasing core energy 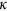, since it becomes more energetically costly to create dislocation pairs. A microscopic determination of 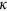 for the stripe phase is unfortunately very complicated and beyond the scope of the present paper. We have therefore adopted a pragmatic approach simply choosing the value 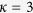 in Fig. 6, which is intermediate between the values of two microscopic models: for the XY model on a lattice one has 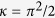
52, whereas BCS theory yields 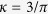 for the 2D superfluid transition, if one equates the core energy with the loss of condensation energy inside a radius given by the BCS coherence length^53^,^54^.

### Melting of supersolid phase

The system exhibits *p*-wave pairing for 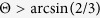
20, which can coexist with stripe order for 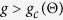 at 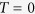
21. We now determine the critical temperature 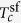 for the superfluid transition. The 2D superfluid transition is in principle also determined by the BKT mechanism, where the topological defects are now vortices. For weak pairing, however, the mean-field BCS theory in fact gives a good estimate of the transition temperature. We thus determine the critical temperature by solving the linearised gap equation


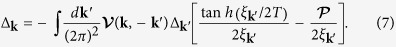


Here 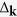 is the gap parameter and 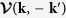 is the effective interaction between the quasiparticles in the stripe phase with energy dispersion 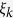 measured from the Fermi surface. The details of this calculation are given in the methods section. The critical temperature obtained from this calculation is shown in Fig. 6 for 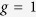 and for several tilting angles. This mean-field result gives an upper bound to the critical temperature, but since 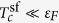 we expect that a more detailed BKT calculation yield only slightly smaller values. This should be contrasted with the melting of the stripes, where an estimate of the critical temperature from a vanishing stripe order would give a much higher value compared to the BKT calculation. This can be seen from Fig. 3, which shows that the mean-field elastic coefficients remain large up to 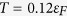. Thus, it is crucial to use the BKT theory to analyse the stripe melting.

Using a simple *p*-wave ansatz for the gap parameter 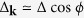, where 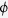 is the polar angle of the wave vector **k**, one can obtain an approximate solution for the critical temperature as


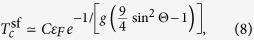


where C is a constant related to an effective momentum cutoff in the integral in (7). We find that the data obtained from solving (7) numerically are in fact very well described by (8) with 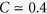.

### Quantum nematic phase for Θ = 0

Figure 6 shows that the critical temperature for the stripe phase vanishes as 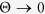. This is a direct consequence of the rotational symmetry rendering 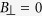 for 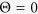. In this case, the system is no longer described by the XY model. Instead, an appropriate expression for the elastic energy of stripe fluctuations is^55^


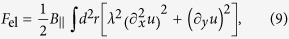


where *λ* is a length comparable to the stripe spacing. Dislocations again play an important role in determining the finite temperature properties of the system described by (9). In contrast to the 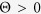 case, however, single dislocations now have a finite energy and can be thermally excited. When the presence of the free dislocations is taken into account, a system described by (9) is predicted to be in a nematic phase for 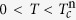, and in an isotropic liquid phase for 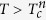
55. In the nematic phase, the translational order exists only within a length scale 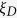, which is determined by the density of the free dislocations. The stripe orientations, averaged over the length scale 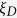, are however algebraically correlated. As a crude physical picture, one can think of the nematic phase as blobs of stripe order of area 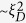, which are all oriented more or less in the same direction, but which are not positionally correlated with each other. The nematic phase is in this sense analogous to the 2D hexatic phase of a crystal, which exhibits bond orientational order but no long-range translational order^47^,^56^,^57^. A quantum hexatic phase was recently predicted to exist in 2D dipolar gases for very strong coupling 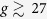
22,23. The results presented here point out the intriguing possibility to realise a quantum version of the nematic phase with dipoles for smaller coupling strengths. We expect the critical temperature 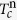 for the melting of the quantum nematic phase to scale as 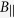. However, a quantitative calculation of the critical temperature for the dipolar system requires knowledge of the parameter *λ*, whose determination is beyond our current theoretical framework. In Fig. 6, we have indicated the critical temperature 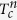 using a somewhat smaller value than the bare 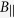 due to renormalisation effects. We note that Fermi surface deformation^58^ leading to a nematic phase^59^ has been predicted for fermionic dipoles in 3D.

## Discussions

An important question concerns whether the critical temperature for the predicted quantum liquid crystal phases is within experimental reach. As an example, let us consider a recent experiment reporting the trapping of chemically stable ^23^Na^40^K molecules in their ground state close to quantum degeneracy. The group obtained an induced dipole moment of 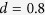 Debye and a maximum 3D density of 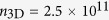 cm^−3^^10^. Estimating a corresponding 2D areal density as 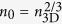, these values correspond to 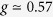. This coupling strength can be increased by reaching a larger fraction of the permanent electric dipole moment of ^23^Na^40^K, which is 2.72 Debye^60^, or by increasing the density of the gas. Since the critical temperature for the nematic and the stripe phases both scale as 
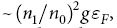
, this indicates that the quantum liquid crystal physics discussed in this paper is within experimental reach, once dipolar gases can be cooled down significantly below their Fermi temperature.

The formation of stripe and superfluid order can be observed as correlation peaks in time-of-flight (TOF) experiments^21^,^42^. One can also detect the stripes directly as density modulations, either after TOF or *in situ*, provided that the experimental resolution is sufficiently high. Observing the proliferation of dislocations would directly confirm the *microscopic* mechanism behind the BKT transition.

An interesting question is how the presence of a harmonic trapping potential in the *xy* plane will influence the results presented here. In the case of a 2D superfluid Fermi gas, recent experiments combined with Monte-Carlo simulations show that the BKT transition survives the presence of a harmonic potential^41^. The exponent 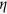 describing the power law decay of the correlation function 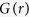 was however found to be significantly larger than its value for a homogenous system. We speculate that the same will be the case for the striped system considered here, since the elastic constants scale as 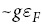 and therefore decrease with decreasing density, so that a trap average will lead to a larger 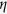. We note that the present system allows for the measurement of the local stiffness constants near the centre of the trap where the system is nearly homogenous, simply by observing the local stripe fluctuations if the experimental resolution is sufficiently high. Alternatively, one can avoid the complications due to a harmonic trapping potential altogether by implementing the box shaped potentials, which have recently been realised experimentally^61^,^62^.

Finally, we would like to mention a recent fixed note Monte-Carlo calculation which suggests that the stripe phase is not the ground state for 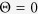 for any coupling strength^24^. We speculate that this result, which contradicts that of refs 14, 15, 16, 17, 18,19,42, is due to the approximate nature of the calculation combined with the fragility of the striped phase, which melts at any non-zero temperature for 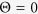, as shown by our results.

In summary, we analysed the phase diagram of a 2D dipolar gases, which exhibits stripe, nematic and supersolid phases corresponding to the breaking of translational, rotational and gauge symmetry. For a non-zero tilting angle 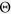, the low energy degrees of freedom of the striped phase are described by an anisotropic 2D XY model. We calculated the stiffness constants corresponding to a rotation and a compression/expansion of the stripes microscopically. This should be contrasted with electron systems, where such stiffness constants are often simply unknown parameters of the theory. The stripes were shown to melt via the Berezinskii-Kosterlitz-Thouless mechanism due to the proliferation of dislocations, and we obtained the melting temperature by solving the relevant renormalisation group equations. We also calculated the critical temperature of the supersolid phase. For 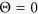, the striped phase is stable only at 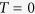, which melts into a nematic phase for arbitrarily small temperatures. Our analysis of the melting temperatures demonstrated that these phases should be within experimental reach. An observation of them would constitute a major breakthrough in our understanding of the interplay between liquid crystal and superfluid order in low-dimensional many-body systems.

## Methods

### Mean-field theory of stripe formation

The mean-field Hamiltonian that takes into account the possibility of stripe formation with a wave vector *q* is given by^42^


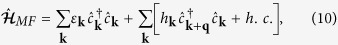


where 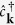 creates a dipole with momentum **k**, 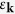 is the single particle Hartree-Fock energy


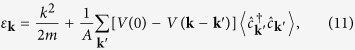


and 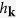 is a real off-diagonal element defined by


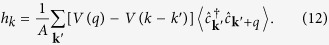


**Figure 7:**
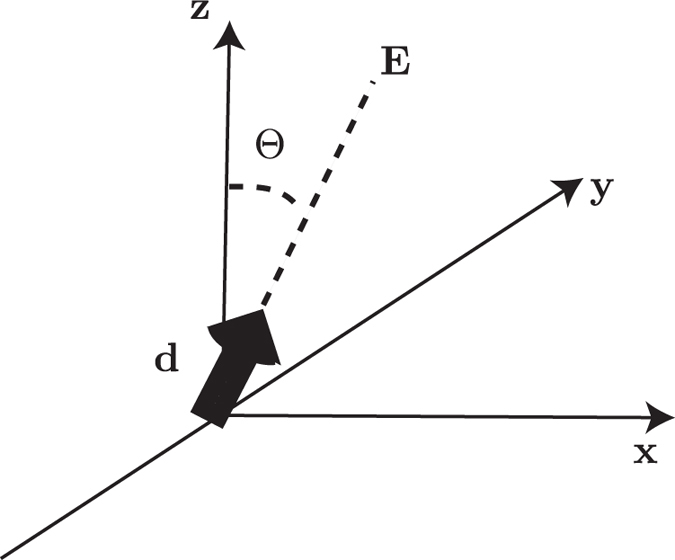
Orientation of the dipole moment. The dipole moment d, aligned by the external *E* field, is perpendicular to the *y*-axis and forms an angle 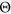 with the *z*-axis.

The quasi-2D interaction in Fourier space is obtained by averaging the interaction over the harmonic oscillator ground state in the *z* direction. This gives (up to an irrelevant constant term)^63^


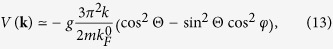


where 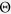 is the orientation angle of the dipole (see Fig. 7) and 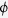 is the polar angle of *k*. We diagonalise the mean-field Hamiltonian by generalising the method described in refs 21,42 to an arbitrary stripe vector *q*. This yields the Hamiltonian


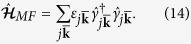


Here 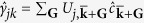 annihilates a quasiparticle with energy 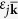, where 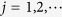 is the band index, 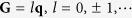 is the reciprocal lattice vector and 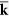 is restricted to the first Brillouin zone of the 1D periodic potential set up by the stripes. We can then calculate the mean-field free energy as 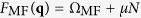, where 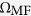 is the mean-field thermodynamic potential given by (5) and 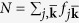 with 
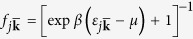
. The interaction energy is most easily calculated using


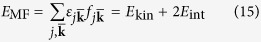


where 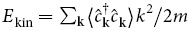 the kinetic energy.

### Renormalisation group equations

We calculate the softening of the effective stiffness constant 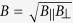 due to the excitations of dislocation pairs using the well-known renormalisation group equations


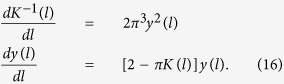


Here 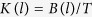 and 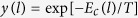 are the scale-dependent stiffness constant and dislocation fugacity respectively. They both decrease with increasing *l* as the renormalisation due to dislocation pairs at larger length scales are included via the solution of (16). The initial values of 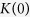 and 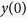 are the bare (local) values unrenormalised by dislocation pairs, which we calculate microscopically as described in the text. At a critical temperature 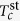, the long range renormalised elastic coefficient 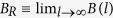 drops to zero by a sudden jump of 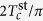. This disappearance of elastic rigidity signals the melting of the stripes.

### BCS theory of the superfluid transition

To explore superfluid pairing within the stripe phase, we use BCS theory with the quasiparticle Hamiltonian 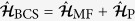. Here,


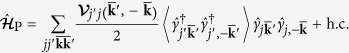


describes pairing between the time-reversed quasiparticles, interacting via


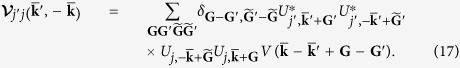


To derive a gap equation that is amenable to a partial wave expansion, we switch to the “extended zone scheme", whereby a single particle state 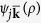 in the *j*’th band in the first BZ is mapped onto a state 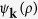 in the *j*’th BZ in the standard way^21^, where the vector **k** is now unrestricted. The effective pairing interaction 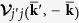 shall be denoted by 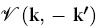 and quasi-particle dispersion 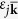 by 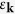. Pairing between time-reversed quasiparticles gives rise to the gap parameter 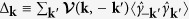, which satisfies the finite temperature gap equation


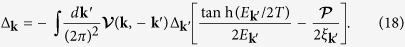


Here 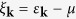 and 
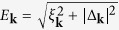
, where the chemical potential *μ* is approximated by the value in the stripe phase. The Cauchy principal value term 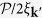 in (18) renders the gap equation well defined with no need for a high momentum cut-off. At temperatures in the vicinity of the superfluid transition, the linearisation of the above gap equation yields (7) we use in the main text. Equation (7) can be solved by the method of partial wave expansion described in ref. 21. Finally we determine the transition temperature by gradually increasing *T* in the gap equation until it ceases to admit finite solutions.

## Additional Information

**How to cite this article**: Wu, Z. *et al*. Liquid crystal phases of two-dimensional dipolar gases and Berezinskii-Kosterlitz-Thouless melting. *Sci. Rep*. **6**, 19038; doi: 10.1038/srep19038 (2016).
